# Entities of Chronic and Granulomatous Invasive Fungal Rhinosinusitis: Separate or Not?

**DOI:** 10.1093/ofid/ofy228

**Published:** 2018-09-14

**Authors:** Ling-Hong Zhou, Xuan Wang, Rui-Ying Wang, Hua-Zhen Zhao, Ying-Kui Jiang, Jia-Hui Cheng, Li-Ping Huang, Zhong-Qing Chen, De-Hui Wang, Li-Ping Zhu

**Affiliations:** 1 Department of Infectious Diseases, Huashan Hospital, Fudan University, Shanghai, China; 2 Pathology Department, Huashan Hospital, Fudan University, Shanghai, China; 3 Department of Otolaryngology, Eye and Ear, Nose and Throat Hospital, Fudan University, Shanghai, China

**Keywords:** chronic invasive fungal rhinosinusitis, classification, granulomatous invasive fungal rhinosinusitis

## Abstract

**Background:**

Chronic and granulomatous invasive fungal rhinosinusitis are important causes of blindness and craniocerebral complications. However, the classification of these 2 diseases remains controversial.

**Methods:**

We retrospectively analyzed patients with chronic and granulomatous invasive fungal rhinosinusitus in a Chinese tertiary hospital from 2009 to 2017, with a focus on classification and comparisons.

**Results:**

Among 55 patients enrolled in our study, 11 (11/55, 20%) had granulomatous invasive fungal rhinosinusitis (GIFRS) and 44 (44/55, 80%) had chronic invasive fungal rhinosinusitis (CIFRS). *Aspergillus fumigatus* and *Dematiaceous hyphomycetes* were identified in 2 patients with GIFRS. Compared with granulomatous type, CIFRS was more frequently encountered in immunocompromised patients (*P* = .022), and the time from onset to diagnosis was much shorter (*P* = .001). Proptosis and orbital apex syndrome showed no significant difference between granulomatous and CIFRS in our study. The treatment options and prognosis of both diseases also showed no significant difference.

**Conclusions:**

Despite the consensus on histopathology, the classification of the chronic and granulomatous types may need further evaluation in clinical considerations.

Fungal rhinosinusitis, an uncommon disease, has been recognized and reported with increasing frequency in recent years [1]. Though much debate exists on its classification, the most commonly accepted system divides fungal rhinosinusitis broadly into 2 groups: invasive and noninvasive according to the histopathological findings. Invasive fungal rhinosinusitis comprises 3 subcategories: acute invasive, chronic invasive, and granulomatous [2]. The acute form has been well described by a disease course of less than 1 month and predominant progressive vascular invasion. Immunocompromised hosts including those with neutropenic or poorly controlled diabetes mellitus are especially vulnerable to acute invasive fungal rhinosinusitis [3]. Aggressive and urgent surgical debridement combined with antifungal treatment is suggested; however, the prognosis is usually extremely poor if the host’s immune status cannot be improved.

Compared with the acute invasive form, chronic invasive fungal rhinosinusitis (CIFRS) and granulomatous invasive fungal rhinosinusitis (GIFRS) are rare. Long and indolent clinical courses usually lasting for more than 12 weeks and even decades are typically reported. Severe complications are usually found as initial manifestations, including blindness, cranial infections, and even death. Therefore, increased attention has been paid to CIFRS and GIFRS, and a differentiation of these somewhat overlapping syndromes and disparate classifications is required.

## METHODS

### Study Design and Patient

This was a retrospective observational study conducted from January 2009 to August 2017 at Huashan Hospital, a tertiary hospital in Shanghai, China. All patients at least 18 years of age and diagnosed with proven GIFRS or CIFRS were eligible for inclusion. Patients who were pregnant, who presented with acute onset (a time course of less than 4 weeks), and without complete data were excluded. Data were collected through medical records, including basic characteristics, predisposing factors, symptoms and signs, laboratory investigation results, image findings (including paranasal sinus computed tomography and cranial magnetic resonance imaging findings), pathological results, antifungal regimens, and patient outcomes. This study was reviewed and approved by the local medical ethics committee.

### Definitions

A proven diagnosis of GIFRS or CIFRS was made according to the following pathological diagnostic criteria [4]: (1) GIFRS: This entity was characterized by granulomatous inflammation with considerable fibrosis. The noncaseating granulomas consisted of numerous multinucleated giant cells and fewer epithelioid cells, lymphocytes, plasma cells, eosinophils, and neutrophils; sometimes, they were associated with vasculitis, vascular proliferation, and perivascular fibrosis. Fungal hyphae were usually rare. (2) CIFRS: Histology showed dense accumulations of hyphae, sparse inflammatory reactions, and occasional presence of vascular invasion. Etiological diagnoses were made by morphological features histologically or phenotype characteristics of culture [4, 5]: (1) *Aspergillus*: *Aspergillus* spp. was usually described as thin, hyaline, septate, acute-angle, and dichotomous branching hyphae. Vesicles with conidia could be observed when the fungi were present in cavitary lesions or sinuses. (2) *Zygomycetes*: *Mucor* species produced nonpigmented, wide, thin-walled, ribbon-like hyphae with few septations and right-angle branching. The hyphae could vary in width, appear folded or crinkled, and be sparse or fragmented. (3) *Dematiaceous hyphomycetes*: The hyphae tended to be thin but were irregularly swollen with prominent septa that showed constrictions and terminal or intercalated vesicular swellings with thick walls resembling chlamydoconidia. Naturally pigmented brown-black hyphae and yeast-like cells structures could be found with hematoxylin and eosin stains. GMS and PAS stains can be used to highlight the fungal wall. Predisposing factors included autoimmune diseases, the use of corticosteroids and immunosuppressive agents, radiotherapy and chemotherapy for malignant tumors, solid organs and hematological malignancies, diabetes mellitus, liver cirrhosis, chronic kidney diseases, solid organ transplant recipients, and immunocompromised states such as idiopathic CD4+ T-lymphocytopenia. In addition, damage or trauma of head-facial structures was also considered a risk factor.

### Statistical Analysis

Statistical analyses were performed with SPSS statistical package, version 22.0. Continuous variables of normal distribution were expressed as mean ± standard deviation (X ± s), and variables of abnormal distribution were shown as median and range. Categorical variables were analyzed using the chi-square (χ^2^) test or Fisher exact test, as appropriate. A *P* value of <.05 was considered statistically significant.

## RESULTS

### Patient Demographics

**Figure 1.**
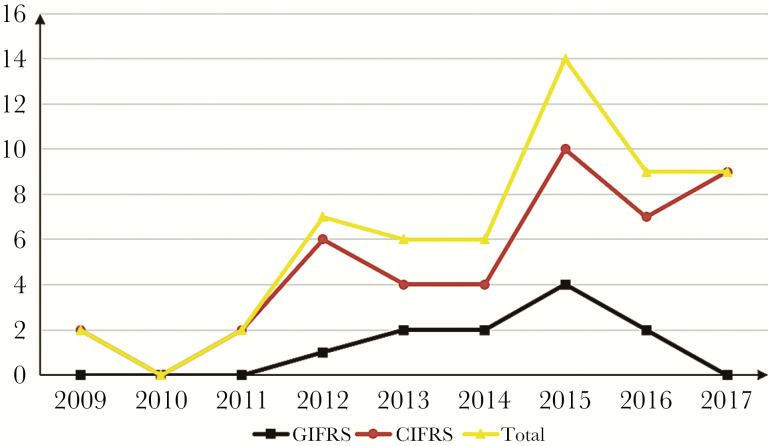
Number of GIFRS and CIFRS cases diagnosed per year. Abbreviations: CIFRS, chronic invasive fungal rhinosinusitis; GIFRS, granulomatous invasive fungal rhinosinusitis.

Overall, 55 patients were enrolled in this study. Seventeen (30.9%) patients were admitted from 2009 to 2013, and another 38 from 2014 to 2017. The number of cases diagnosed tended to increase each year (Figure 1). Thirty-two of the patients were male and 23 were female, and the median age was 57 years. The demographic and clinical characteristics of 11 GIFRS and 44 CIFRS patients are shown in Table 1.

**Table 1.** Comparison of Baseline and Clinical Characteristics of Patients With GIFRS and CIFRS

Characteristics	Patients With GIFRS (n = 11)	Patients With CIFRS (n = 44)	*P* Value
Age, median (range), y	46 (38–71）	57 (25–88)	NS
Male	9 (81.8)	23 (52.2)	NS
Immunocompromised factors	0 (0.0)	16 (36.3)	.023
** **Type 2 diabetes mellitus	0	9	NS
** **Solid organ tumors	0	5	NS
** **Autoimmune diseases	0	4	NS
** **Cirrhosis	0	1	NS
Trauma or destructions of facial structures	1 (9.1)	7 (15.9)	NS
Manifestations			
** **Nasal symptoms	3 (27.3)	23 (52.3)	NS
** **Ocular symptoms	7 (63.6)	26 (59.1)	NS
** **Ear symptoms	2 (11.1)	2 (4.5)	NS
** **Facial symptoms	5 (45.5)	24 (54.5)	NS
** **Headache or dizziness	5 (45.5)	29 (65.9)	NS
** **Nausea and vomiting	0 (0.0)	6 (13.6)	NS
** **Systematic symptoms	0 (0.0)	9 (20.4)	NS
** **Conscious disturbance	0 (0.0)	3 (6.8)	NS

Data are presented as No. (%) unless otherwise indicated.

Abbreviations: CIFRS, chronic invasive fungal rhinosinusitis; GIFRS, granulomatous invasive fungal rhinosinusitis; NS, no significance.

Of the 55 patients, 11 (20.0%) were identified with GIFRS, whereas 44 were diagnosed with CIFRS. One or more predisposing factors were found in 24 patients (43.6%). The most frequent underlying disease was type 2 diabetes mellitus (n = 9), followed by solid organ tumors (n = 5), autoimmune diseases (n = 4), and cirrhosis (n = 1). Five of the above-mentioned patients were co-administered immunosuppressants or steroids, whereas 2 patients received radiotherapy. Eight patients experienced trauma or destruction of facial structures.

### Clinical Manifestations and Radiological Presentations

Head and paranasal computerized tomography and enhanced magnetic resonance imaging were performed in all patients, as shown in Table 2. The abnormalities included thicker mucosa, soft tissue density, anf formation of granuloma, masses, and abnormal signals; no significant difference was found. The most prominent manifestations included headache and dizziness (34/55, 61.8%), ocular symptoms (32/55, 58.1%), and facial symptoms (28/55, 50.9%). Other symptoms included systematic symptoms, such as fever, consciousness change, limb dyskinesia, nausea and vomiting, altered consciousness, toothache, and mastoid tenderness.

**Table 2.** Comparisons of Involvement and Etiology of GIFRS and CIFRS

Variables	Patients With GIFRS (n = 11)	Patients With CIFRS (n = 44)	*P* Value
Radiology			
** **Lesion enhancement	6 (54.5)	16 (36.3)	NS
** **Bone absorptions or destructions	3 (27.2)	11 (25.0)	NS
** **Orbital or optic nerve abnormalities	3 (27.2)	13 (29.5)	NS
** **CNS radiological abnormalities	5 (45.4)	19 (43.2)	NS
Involvement sites			
Paranasal sinus			
** **Single sinus	2 (18.1)	12 (27.3)	NS
** **Multi sinus	9 (81.8)	33 (75)	NS
** **Frontal sinus	3 (27.3)	12 (27.3)	NS
** **Sphenoid sinus	6 (54.5)	30 (68.2)	NS
** **Ethmoid sinus	9 (81.8)	27 (61.4)	NS
** **Maxillary sinus	8 (72.7)	31 (70.5)	NS
** **Ocular involvement	5 (45.5)	22 (50.0)	NS
** **CNS involvement	5 (45.5)	20 (45.5)	NS
** **Ocular and CNS involvement	3 (27.3)	7 (15.9)	NS
Other sites			
** **Mastoid process	2 (18.1)	3 (6.8)	NS
** **Lung	1 (9.1)	5 (11.4)	NS
** **Facial region	0 (0)	3 (6.8)	NS
Etiology			
** ** *Aspergillus* spp.	9 (81.8)	40 (90.9)	
** ** *Aspergillus fumigates*	1 (9.1)	1 (2.3)	NS
** ** *Candidal albicans*	0 (0.0)	1 (2.3)	NS
** ** *Zygomycete*	0 (0.0)	3 (6.8)	NS
** ** *Dematiaceous hyphomycetes*	1 (9.1)	0 (0.0)	NS
Death	0 (0.0)	6 (13.6)	NS
Symptom onset to diagnosis			
Median time, mo	4	24	.001

Data are presented as No. (%) unless otherwise indicated.

Abbreviations: CIFRS, chronic invasive fungal rhinosinusitis; CNS, central nervous system; GIFRS, granulomatous invasive fungal rhinosinusitis; NS, no significance.

The sites involved in CIFRS and GIFRS are illustrated in Table 2. All patients had nose or paranasal involvement. Forty-two patients were found with multiparanasal involvements; all paranasal sinuses were involved in 3 patients. Concurrent involvement of the maxillary and ethmoid sinuses (n = 27) was also commonly found. Notably, patients with left paranasal sinus lesions (43/55, 78.2%) had a tendency for central nervous system (CNS) or ocular lesions, compared with those with right lesions (35/55, 63.6%). However, no significant difference was found.

### Treatment and Outcome

All patients received antifungal treatment. Voriconazole or itraconazole was administered as initial therapy in 48 patients, and amphotericin B deoxycholate (AmB)–based antifungal treatment was used in 7 patients. The median duration of antifungal therapy was 6 months (ranging from 2.7 to 31 months). Adverse effects were seen in 19 patients. In addition to antifungal therapy, 30 out of 55 (54.5%) patients also underwent surgical intervention. The most common intervention was open debridement of the paranasal sinus (n = 20), followed by local mass excision (n = 15), nasal septal reconstruction (n = 3), functional endoscopic sinus surgery (n = 1), and optic nerve decompression (n = 1).

The median time for follow-up in these patients (range) was 10 (1–18) months. The median time for clinical symptom relief after antifungal treatment (range) was 6 days (1 day–2 months). Except for 2 patients with CIFRS who died of severe pulmonary infection and sepsis, respectively, and 4 due to self-drug withdrawal, 49 patients were free of invasive fungal disease at the last follow-up, with the effective rate being 89.1%.

### Comparison of Clinical Features Between Patients With GIFRS and CIFRS

We analyzed the data of 55 patients. Significant differences were not found in demographic characteristics, clinical profiles, and laboratory findings between the CIFRS and GIFRS groups. Predisposing factors were found in all CIFRS patients, except 1 patient with a history of left tear sac resection. Notably, CIFRS rather than GIFRS primarily affected immunocompromised hosts (*P* = .023). The median time from symptom onset to diagnosis (range) was 4 months (1 month–7 years), and the time of the granulomatous group was much longer than the chronic form (*P* = .001).

All cases were confirmed by histopathological examinations. The culture of the tissue was also conducted in 3 patients; 2 revealed *A. fumigatus,* and 1 was *Candida albicans*. *Aspergillus* spp. (51/55, 92.7%) was the most common pathogen, followed by *Zygomycetes* (3/55, 5.5%), *C. albicans,* and *Dematiaceous hyphomycetes* (1/55, 1.8%). Notably, specimens from 2 patients with GIFRS were identified as *A. fumigatus* and *Dematiaceous hyphomycetes*, respectively, whereas *Zygomycetes* (n = 3), *C. albicans* (n = 1), and *A. fumigatus* (n = 1) were found in patients with CIFRS. The comparisons of etiology are summarized in Table 2.

The 2 groups showed no significant difference in survival curve (*P* = .225), and prognosis for GIFRS did not improve, though CIFRS was considered a more aggressive disease.

## DISCUSSION

Early in 1997, DeShazo et al. proposed a new classification of invasive fungal rhinosinusitis: acute (fulminant) invasive, granulomatous invasive, and chronic invasive types [6]. Since then, there has been a continued debate on the classification system. In 2009, a consensus that the CIFRS and GIFRS forms had enough clinical and pathological differences to be classified as separate entities was reached [4]. However, the classification remained controversial [7].

 In this descriptive study of 11 GIFRS and 44 CIFRS patients, all were diagnosed by histopathology. Our result found significant differences in immune status and the median time from onset of symptoms to diagnosis. All patients diagnosed with GIFRS were immunocompetent in our study; meanwhile, 55.5% of CIFRS patients had predisposing factors (*P* = .023), which was consistent with some recent studies that the granulomatous type was frequently encountered in immunocompetent patients. Immunocompromised patients were more susceptible to CIFRS, as histopathological differences might be a function of manifestation of the immune status [8]. Though both types are insidious and chronic diseases, our study also showed a significant difference in the median time from onset of symptoms to diagnosis (*P* = .001), possibly because of the indolent presentation of granulomatous forms. The median time from onset of symptoms to diagnosis was 4 and 24 months for CIFRS and GIFRS patients, respectively.

 Despite such differences between the 2 groups, we have not found any significant differences between granulomatous and chronic groups in clinical settings. Multi-involvement and complications are commonly encountered in both groups. Cranial neuropathy, visual loss, and orbital pain were the most common complications encountered in cases of fungal rhinosinusitis, according to Cho et al. [9]. Similarly, headache, dizziness, and visual loss were the most common complications in our patients, and orbital involvement is a feature of both types of fungal rhinosinusitis. Nearly half of our patients had ocular radiological involvement, with an incidence of 46.5% in CIFRS and 45.5% in GIFRS, respectively. Higher prevalence rates are reported in the literature. Studies of invasive fungal rhinosinusitis cases had shown a prevalence of clinical evidence of orbital involvement of up to 100% in some series [10–12]. Of note, concurrent involvement of ocular and CNS systems was found in 7 CIFRS patients and 3 GIFRS patients, and 6 patients died in our study. As these diseases are associated with severe complications and poor prognosis, patients with these diseases should be monitored carefully.

As for treatment or prognosis, Busaba et al. reported that the differences between the 2 groups were of little importance with respect to choosing a therapy or determining the prognosis, which should be based on the extent of the disease at the initial diagnosis and its subsequent clinical course [13]. Panda et al. divided fungal rhinosinusitis into acute and chronic invasive categories for the following reasons: similar histopathological evidence of mucosal invasion, a fixed treatment protocol for both diseases, and similar clinical manifestations, such as involvement of multiple sinuses and orbital or intracranial involvement [14]. We reported 6 deaths in 55 cases, all found in the CIFRS group. However, the survival rate of the 2 groups assessed via the Kaplan-Meier method had no significant difference. Panda et al. reported no recurrence after 1 year of follow-up in 6 chronic invasive cases [14]. D’Anza et al. also found no recurrence in a study of 6 patients with CIFRS [15]. The literature investigating the treatment of CIFRS is comprised almost exclusively of retrospective case series, and there is no consensus on the best treatment [16]. Most authors have recommended antifungal treatment and endoscopic debridement of necrotic sinonasal tissue, whereas some authors believe that the reduction of immune suppression when feasible also plays a role, as in chronic granulomatous diseases, where adjuvant interferon has been shown to significantly reduce infections [17, 18]. The initial option of antifungal agent depends on the etiology [19]. However, regardless of the therapy administered, the mortality and prognosis seemed no different between these 2 types, and early diagnosis and timely antifungal treatment were critical for the improvement of the prognosis.

Granulomatous invasive fungal rhinosinusitis (GIFRS) used to be considered a separate species and has been primarily described in the Sudan, India, and Pakistan and occasionally found in America, indicating that it may be a geographical or ethnicity-related entity [20–24]. However, 11 patients in our study were diagnosed with GIFRS, suggesting that the granulomatous form is not unusual in China. Of note, *A. fumigatus* and *Dematiaceous hyphomycetes* were identified in 2 GIFRS separately, which was not consistent with some studies reporting that GIFRS was infected almost exclusively with *A. flavus* [25].

However, proptosis, which is considered a typical feature in GIFRS patients, was first described in the Sudan by Milosev et al. in 88.2% of patients with granulomatous forms [4, 26]. Chandrasekaran et al. reported that 77% of patients with GIFRS presented with proptosis, Veress et al. reported 60.8%, and Dhiwakar et al. reported 67.7% [27]. Nevertheless, Panda et al. found that proptosis had a comparable frequency among patients with noninvasive aspergillus sinusitis (41.6%) [14]. Our data showed a similar prevalence of 27.2% in GIFRS cases and 18.6% in CIFRS cases, indicating that proptosis might not be a GIFRS-exclusive symptom. Meanwhile, orbital apex syndrome used to be regarded as a classic manifestation of CIFRS that usually affects immunocompromised patients. Orbital apex syndrome is characterized by decreased vision and ocular immobility resulting from a mass in the superior portion of an orbit [28–30]. Though the prevalence of orbital apex syndrome of CIFRS (24/44, 54.5%) was higher than that of GIFRS (5/11, 45.5%), our study has not found a significant difference (*P* = .739). Similarly, Challa et al. reported 8 of 10 CIFRS (80%), whereas 10 of 19 (52.6%) in the GIFRS group presented with orbital apex syndrome; there was no significant difference (*P* = .234), indicating that proptosis and orbital apex syndrome were not obviously characteristics in GIFRS and CIFRS, respectively [31].

Even if GIFRS was more frequently encountered in immunocompetent patients with much longer time from onset of symptoms to diagnosis, the treatment and prognosis of CIFRS and GIFRS have no significant differences. Both indolent diseases are invasive and commonly involved CNS and ocular sites. Especially in our study, *A. fumigatus* and other rare fungi were isolated in granulomatous categories, whereas proptosis and orbital apex syndrome were frequently found in both groups, indicating that the classification of CIFRS and GIFRS may not play an important role in the clinical setting. However, owing to the small number of subjects and the retrospective nature of the study, additional data are needed to corroborate our findings.
